# Development and validation of the cognitive flexibility scale for AI-supported English language learning (CFS-AISEL)

**DOI:** 10.3389/fpsyg.2026.1776879

**Published:** 2026-08-28

**Authors:** Xiaomeng Li, Huan Li

**Affiliations:** 1 School of Educational Studies, Universiti Sains Malaysia, Penang, Malaysia; 2 School of Foreign Languages, Qiongtai Normal University, Haikou, Hainan, China

**Keywords:** AI-supported English learning, cognitive flexibility, evaluation methodologies, human-computer interface, scale development

## Abstract

The widespread use of AI technology in English language learning and instruction presents numerous challenges for EFL learners, which require higher levels of cognitive flexibility to address. However, existing cognitive flexibility questionnaires are insufficient to comprehensively reflect changes in cognitive flexibility within AI-supported learning environments. Therefore, this study developed the Cognitive Flexibility Scale for AI-Supported English Language Learning (CFS-AISEL) based on a systematic literature review and rigorous questionnaire development methodology. A random sampling method was employed to collect 1,093 responses for exploratory factor analysis (EFA) and confirmatory factor analysis (CFA). Of these, 479 were used for EFA and 614 for CFA. A Delphi study was applied for scale content validation. After confirming reliability and validity, the final scale comprised 26 items across five dimensions (cognitive flexibility, tolerance for ambiguity, self-efficacy in AI interactions, emotional flexibility, and ethical flexibility). This study provides practical implications for educators to identify how EFL learners balance the use of AI tools and independent thinking, promoting the deep integration of educational assessment and AI technology.

## 1 Introduction

With the development of artificial intelligence (AI), the landscape of language learning has undergone a revolutionary transformation. By offering a low-pressure environment, personalized feedback, and flexible learning opportunities, AI technologies—particularly conversational AI—can reduce stress, boost motivation and confidence, and ultimately reshape both educational paradigms and learner–technology relationships (Ji et al., 2023).

In technology-enhanced education, cognitive flexibility refers to learners’ capacity to switch between different interpretive frames or problem-solving strategies when interacting with adaptive learning systems or dynamic task environments (Diamond, 2013; Wang and Jou, 2023). It is an essential tool for managing demanding and dynamic AI learning environment (Chauncey and McKenna, 2023; Egner and Siqi-Liu, 2024), which can be influenced by culture and helps learners overcome cognitive rigidity and resistance to change. In addition to these observations, Chauncey and McKenna (2024) demonstrated how cognitive flexibility in English as a Foreign Language (EFL) instructors can increase creativity and critical thinking abilities. Collectively, these scholarly contributions converge on a compelling narrative: cognitive flexibility represents a critical psychological mechanism that not only facilitates linguistic proficiency but also contributes substantively to broader cognitive and pedagogical outcomes.

### 1.1 Problem statement

While cognitive flexibility demonstrates significant potential in language learning, the emerging scholarly discourse has illuminated the intricate problems introduced by AI (Na-songkhla et al., 2024; Mohebbi, 2025). Recent empirical studies have broadened the scope of research into philosophical dexterity and examined the challenging issues raised by human-AI relationships. Samuel et al. (2022) stated that users are required to adapt their decision-making processes and create new strategies for navigating complex and evolving environments due to the various information facets and cognitive challenges brought about by AI, such as rapid information processing and increased cognitive load. In this context, executive functions, including cognitive flexibility, are crucial because they help people manage more complex cognitive demands posed by scientific interactions (Friedman et al., 2016; Turan et al., 2024). This discourse was amplified by Ozmen Garibay et al. (2023), highlighting pressing social issues such as cultural barriers and the development of nuanced interpersonal dynamics in technological interactions. According to research, the main problems are social dilemmas, less confidence in AI algorithmic decision-making, and potential misuse of shared information (Ozmen Garibay et al., 2023; Sullivan and Fosso Wamba, 2022). Cognitive flexibility emerges as a crucial tool for navigating these challenging situations because it enables people to support their critical thinking by thoroughly examining social implications from various perspectives and critically examining AI’s ethical, social, and cultural effects, thereby promoting individual adaptability and wellbeing (Fuchs et al., 2024; Orakcı and Khalili, 2025). Despite significant insights into cognitive flexibility’s role in language learning and its connections with AI, substantial scientific and philosophical questions remain unanswered regarding how cognitive flexibility operates specifically within AI-supported language learning conditions. In order to understand the cognitive processes that emerge from technological interactions, more advanced systems must be used to comprehend the complex relationships among academic advancement, language acquisition, and cognitive flexibility. In order to bridge the current research gaps (how cognitive flexibility specifically operates in AI-supported language learning conditions remains unanswered) and provide a reliable analytical tool for upcoming studies, this study aims to create a valid cognitive flexibility scale for AI-supported language learning situations.

## 2 Literature review

### 2.1 Conceptualization of cognitive flexibility

Cognitive flexibility is adapting cognitive processing strategies in response to novel and unexpected environmental conditions (Cañas et al., 2003). This adaptive capability enables individuals to shift between concepts, integrate multiple perspectives, and modify behaviors and thought patterns accordingly (Dennis and Vander Wal, 2010). The concept of cognitive flexibility emerged from educational psychology and cognitive science in the late 20th century with its foundational development tied to cognitive flexibility theory (CFT) (Spiro et al., 2003), which defines it as the capacity to adapt cognitive processes across diverse contexts. In the late 20th century, research went beyond simply receiving appropriate training to acquire its importance in more general social and psychological circumstances. Martin and Rubin (1995) identified cognitive flexibility as a vital aspect of communication competence, supporting perspective-taking, behavioral adaptation, and self-efficacy in making proper options, highlighting its importance in interpersonal adaptability. In addition to advocating for diverse knowledge representation and technological integration to foster adaptability, Hernandez-Serrano et al. (2000) emphasized its role in constructivist learning, problem-based learning, and technology-enhanced education.

Recent studies (Diamond and Ling, 2020; Miyake et al., 2000) examine cognitive flexibility as a component of cognitive control. People can regulate thoughts, switch ideas, and adapt to new learning circumstances. The Cognitive Flexibility Inventory (CFI), as used by Dennis and Vander Wal (2010), significantly influences psychological resilience and emotional regulation. The CFI comprises two key subscales: (a) perceived alternatives, which assesses the ability to generate multiple explanations for events; and (b) control, which measures the ability to manage difficult situations and restructure dysfunctional thought patterns. Diamond (2013) expanded on this by defining cognitive flexibility as a fundamental executive function (set-shifting), highlighting its crucial role in adaptability learning and problem-solving. Ionescu (2012, 2017) reconceptualized cognitive flexibility as a dynamic system property driven by multiple interactive mechanisms that respond to environmental demands while Martin and Rubin (1995) as well as Dennis and Vander Wal (2010) examined cognitive flexibility in applied contexts such as communication and psychological resilience. This framework views cognitive flexibility as a whole-system agility rather than a task-shifting capacity, particularly during uncertainty and difficulty, thus challenging conventional measurement methods. Besides, cognitive flexibility includes perspective shifting, which enables learners to adopt multiple points of view and reinterpret information in response to contextual demands (Diamond, 2013; Dumontheil et al., 2010).

Grounded in CFT (Spiro et al., 1988) and established neurocognitive frameworks (Dennis and Vander Wal, 2010; Miyake et al., 2000), core cognitive flexibility refers to the executive ability to shift mental sets, adapt processing strategies to changing task demands, and concurrently consider multiple conceptual perspectives. Crucially, this core construct is distinct from: (a) adaptability (an outcome manifested through behavioral adjustment), (b) emotional regulation (managing affective states), and (c) ethical reasoning (weighing moral principles). Early research on cognitive flexibility primarily focused on task-switching and cognitive control (Miyake et al., 2000; Monsell, 2003). However, the conceptualization has since broadened to encompass domains such as emotional regulation, communication adaptability, and adaptable functioning across various contexts, for instance ecological systems (e.g., Dennis and Vander Wal, 2010; Martin and Rubin, 1995). These conceptual developments highlight cognitive flexibility as a dynamic, context-dependent construct essential for navigating ambiguous and challenging situations. Building on this tradition, the present study further extends cognitive flexibility by contextualizing it within AI-supported English language learning, leading to a multidimensional framework that includes tolerance for ambiguity, AI-interaction self-efficacy, emotional flexibility, and ethical flexibility alongside core cognitive flexibility.

### 2.2 The role of cognitive flexibility in AI-supported English learning environment

Cognitive flexibility demonstrates its importance in language acquisition working through many pathways. It has been noted that language learners face different cognitive challenges as they have to comprehend and integrate different cultural practices in the language (Zhai and Wibowo, 2023). This flexibility facilitates complex language system navigation and communication skill refinement across different settings (Diamond and Ling, 2020). This cognitive flexibility also supports a dynamic switching process in language learning, enabling a learner to switch from one language to another with ease (Johann et al., 2020), boosts other aspects of control (Bialystok and Craik, 2010; Green, 1998), and aids in the synthesis of knowledge (Prior and MacWhinney, 2010). The effects of cognitive flexibility are observed in various language learning processes such as in interpreting (Togato and Macizo, 2023), reading (Barber et al., 2020; Huang et al., 2022), and writing (Perpiñà Martí et al., 2023). Cognitive flexibility operates in relation to other psychological factors (including motivation, working memory, and anxiety), which in turn affects the level of academic achievement (Wang and Jou, 2023). Cognitive flexibility’s contribution to problem-solving and multitasking skills by reducing task-switching costs and improving dual-task performance is highlighted by Strobach et al. (2012). This in-depth analysis stresses cognitive flexibility as a significant factor in developing independent and flexible language learners.

A connection exists between AI technology and language learning as evidenced by the integration of AI tools into language learning research methodologies. Drawing on cognitive and educational theories, recent studies suggest that AI may support the development of cognitive flexibility through personalized and adaptive learning environments (Abulibdeh et al., 2024; Muthmainnah et al., 2024; Zhai and Wibowo, 2023). Drawing on Spiro et al.’s (2003) cognitive flexibility theory and Ionescu’s (2012) multidimensional processing framework, AI-supported language learning platforms enable learners to continuously reinterpret linguistic knowledge, offering a key advantage over traditional direct instruction methods. This continuous process of knowledge reinterpretation encourages students to create exceptional mental strategies that transcend data transfer. Moreover, to mitigate the emotional strains that could impede such deep cognitive processing, recent evidence (Chen et al., 2021) highlights the importance of supportive learning environments. Rodríguez-Ardura and Meseguer-Artola (2016) consistently demonstrated how AI systems create multiple knowledge representations and contextual variations, and Wu (2023) further emphasizes that beyond this representational and contextual diversity, cognitive flexibility serves as a key skill for overcoming learning-related barriers and improving information processing in language acquisition. The transition from receptive to active learning arguably represents one of the most notable developments in the field. Chauncey and McKenna (2023) and Wang et al. (2023) emphasized that deep, reflective engagement with AI tools creates a more personalized, adaptive learning community. In particular, large language models (LLM) help students navigate challenges with unparalleled independence and effectiveness (Woo et al., 2026). Further, Shin (2024) identified three mechanisms of cognitive flexibility in AI-enhanced learning: processing AI feedback, regulating emotions during technology interaction, and making ethical decisions when using AI tools. Namaziandost and Rezai (2024) built on this three-dimensional framework by demonstrating the interconnection between ethical issues and learners’ emotional responses to AI feedback.

The integration of AI in educational contexts delivers multifaceted advantages, though its distinctive pedagogical innovations coexist with nontrivial systemic risks. Pioneering research identifies multiple risks associated with excessive AI dependency. Zhai et al. (2024) postulate that such dependency precipitates cognitive erosion, diminishes critical thinking, impairs decision-making abilities, and increases susceptibility to AI-generated errors or hallucinations. Darwin et al. (2024) specifically highlight the erosion of critical thinking. Memarian and Doleck (2023) draw attention to issues of fairness, accountability, transparency, and ethics, and further note that this reliance can lead to higher instances of plagiarism and challenges in responsible content dissemination. Meanwhile, Shin (2024) focuses on the risks of misinformation and algorithmic bias. Empirical findings indicate that cognitive flexibility, through optimizing learners’ adaptability, mediates critical thinking during human-AI co-regulation processes and facilitates systematic evaluation of AI algorithmic outputs, navigation of ethical issues, and cognitive equilibrium between AI support and independent thinking (Fuchs et al., 2024; Orakcı and Khalili, 2025). The confluence of AI and language acquisition reveals intricate emotional dynamics, wherein learners’ technological predispositions (as mediators) significantly shape affective responses within AI-enhanced learning environments, including heightened anxiety, operational frustration, and perceived autonomy depletion, particularly among technologically apprehensive individuals (Priporas et al., 2024; Wang and Hui, 2024). Conversely, learners possessing robust technological self-efficacy manifest enhanced behavioral adaptability, characterized by sustained motivation, cognitive assurance, and task immersion (Bi et al., 2024; Wu et al., 2023). Crucially, the pedagogical efficacy which operationalized as the extent to which AI-driven systems facilitate vocabulary acquisition, grammatical accuracy, and communicative competence (Kaongoen et al., 2023) remains contingent upon learners’ technological receptivity and cognitive elasticity.

In production, the contribution of AI in language learning offers ethical and emotional issues that need augmented cognitive flexibility for effective navigation. With this freedom, individuals can maintain ethical discernment amidst emotional regulation processes, concurrently enabling critical evaluation of AI-generated output and psychosocial adaptation to technological integration.

### 2.3 Limitations of existing cognitive flexibility scales

The Cognitive Flexibility Scale (CFS), developed by Martin and Rubin (1995), assesses three factors: awareness of communication alternatives, willingness to adapt to situations, and self-efficacy in being flexible. By contrast, the Cognitive Flexibility Inventory (CFI) created by Dennis and Vander Wal (2010) comprises two factors: flexible selection and flexible control perception. The flexible selection subscale explains how individuals respond to life events and behaviors by offering multiple solutions across various situations. The flexible control subscale measures individuals’ belief in their ability to manage difficult situations. Both the CFS and CFI strongly emphasize adaptability and tolerance for ambiguity, highlighting the complex link between cognitive flexibility and psychological wellbeing. Additionally, Johnco et al. (2014) compiled the Cognitive Flexibility Questionnaire. This instrument focuses on assessing the general ability to generate psychological alternatives. However, it lacks specific content related to AI-enhanced learning.

Nonetheless, existing questionnaires have significant limitations when applied to AI contexts. Notably, they lack comprehensive consideration of ethical flexibility. Current research on cognitive flexibility in AI environments relies mainly on the CFI developed by Dennis and Vander Wal (Grant and Cassidy, 2022; Tanhan et al., 2024; Wang and Jou, 2023). Although this instrument is effective for measuring personalized emotional regulation, it does not adequately capture the distinctive characteristics of AI-supported learning environments. In such settings, EFL learners must continuously adapt to dynamic, system-generated feedback such as personalized learning recommendations and real-time error corrections (Yang et al., 2024). The instantaneous and evolving nature of this feedback imposes increasingly complex demands on learners’ cognitive flexibility. Critically, the current CFI framework does not sufficiently address these nuanced adaptive requirements, revealing a significant gap in existing cognitive flexibility assessment methodologies.

### 2.4 Theoretical framework

Drawing on cognitive flexibility theory (CFT), insights from the preceding literature review, and existing questionnaire measures—specifically their dimensional structures, item content, and response formats—this study developed the Cognitive Flexibility Scale for AI-Supported English Language Learning (CFS-AISEL), which comprises five potential measurement dimensions: cognitive adaptability, tolerance for ambiguity, self-efficacy in AI interactions, emotional flexibility, and ethical flexibility. The subsequent sections will elaborate the five potential dimensions.

Martin and Rubin (1995) emphasized adaptability as a core component of cognitive flexibility while Ionescu (2012) identified strategic adaptation as a central mechanism. Regarding tolerance for ambiguity, although not explicitly part of traditional cognitive flexibility scales, ambiguity tolerance is critical in interpreting imperfect or probabilistic AI-generated suggestions. CFT also highlights tolerance for ambiguity, making it easier for people to feel more at ease with uncertainty and complexity as well as enhancing people’s ability to navigate situations without formulaic recommendations (Burkolter et al., 2009). Ionescu (2012) noted the importance of flexibility in responding to uncertain or novel demands, and in AI-supported language learning, ambiguity emerges when learners must infer meanings or strategies from incomplete or inconsistent AI feedback. Martin and Rubin (1995) stressed that self-efficacy is a part of cognitive flexibility because even if individuals are aware of alternative behavioral choices in a given situation and are willing to be flexible, they also need to believe in their capacity to effectively implement desired behaviors. Dennis and Vander Wal (2010) discussed cognitive flexibility as critical in managing stress and adapting strategies, which aligns with the need for self-efficacy in navigating AI interactions. CFT focuses on emotional dexterity, which allows for adaptive thinking in different contexts and facilitates knowledge transformation, fostering problem-solving from multiple angles.

Bandura’s (1997) self-efficacy theory underscores confidence’s pivotal role in shaping adaptability within emerging technological landscapes. Emotional flexibility, conceptualized as the modulation of affective reactions to obstacles in AI-integrated learning systems, was empirically connected by Dennis and Vander Wal (2010) to stress mitigation and psychological resilience preservation. This construct proves indispensable when addressing AI-induced disruptions including algorithmic anomalies, protracted skill acquisition phases, or technological saturation. Ethical flexibility denotes the capacity to recalibrate moral frameworks when confronting AI-specific challenges such as data privacy breaches, systemic biases, or intellectual authenticity dilemmas (Deliu and Moldovan, 2026). Grounded in Ionescu’s contextual flexibility paradigm, this dimension specifically targets AI-native ethical quandaries (Ionescu, 2017). Current debates in machine ethics (e.g., Zuboff, 2019) emphasize learners’ imperative to negotiate techno-ethical paradoxes. Accordingly, building upon this theoretical foundation, the research systematically operationalizes each construct as detailed in Table 1.

**TABLE 1:** Definitions of potential factors.

Aspects	Definitions
Cognitive adaptability (CA)	Flexibility in adjusting thinking and learning strategies when engaging with dynamic AI tools.
Ambiguity tolerance (AT)	Ability to handle uncertainty inherent in AI-generated feedback and outcomes.
Self-efficacy in AI interactions (SE)	Confidence in using AI tools effectively for language learning.
Emotional flexibility (EMF)	Regulation of emotional responses to challenges in AI-mediated learning environments.
Ethical flexibility (EF)	Adapting ethical considerations to new challenges posed by AI.

### 2.5 Research gap

Although cognitive flexibility has been studied in conventional educational settings, less is known about how AI and cognitive flexibility connect with language learning. Specifically, there is insufficient instrument to assess cognitive flexibility in AI-supported language learning. The CFI and the CFS have been employed in broad cognitive domains. These psychometric tools demonstrate limited ecological validity when evaluating the sophisticated human-AI interaction dynamics inherent to linguistic skill acquisition environments. There is a need for comprehensive models that incorporate emotional and ethical dimensions when interacting with AI tools for language learning. Thus, to solve these pressing problems and provide a solid measurement tool that effectively assesses the variety of cognitive flexibility in an AI-supported language learning environment, this study creates and tests the CFS-AISEL.

## 3 Methodology

### 3.1 Item development

Drawing upon an extensive review of cognitive flexibility literature and its role in AI-supported English language learning, the initial phase of scale development established a theoretical foundation for item generation. The literature synthesis identified essential components of cognitive flexibility within AI-supported contexts, followed by a systematic analysis of existing cognitive flexibility measures to identify adaptable items. These potential items underwent rigorous examination and modification to align with the specific context of the study. The subsequent phase involved collaborative item generation through a focus group consisting of four experts spanning educational technology and technology-assisted language instruction. The focus group systematically developed items corresponding to the five factors of CFS-AISEL: cognitive flexibility, ambiguity tolerance, self-efficacy in AI interactions, emotional flexibility, and ethical flexibility. The expert consultation process ensured content validity while providing crucial insights into the requisite elements for each dimension. The synthesis of focus group deliberations and literature findings culminated in an initial pool of 30 items, each precisely formulated to capture its respective dimension and scrutinized for clarity and relevance. The instrument employed a 5-point Likert scale (1 = strongly disagree, 2 = disagree, 3 = moderately agree, 4 = agree, 5 = strongly agree), offering respondents a wider range of response options to capture more nuanced variations in their perceptions. This scaling approach was chosen for its increased sensitivity in measuring complex psychological constructs while maintaining reasonable response differentiation (Finstad, 2010).

### 3.2 Delphi validation-A mixed-methods approach

The initial 30-item pool received content validation through expert review. Eight scholars with experience of using AI technology in English instruction and expertise in educational technology were invited to evaluate the items. A comprehensive validation form was developed with explicit review criteria delineated at the outset. The experts were requested to assess and provide feedback on each item, rating them on a 4-point scale across three dimensions: relevance (whether the item reflects the target construct), clarity (whether the item is clear and understandable to the target population), and ambiguity (whether the item contains vague or double meanings that could confuse respondents). In terms of relevance, the score ranges from 1 (Not relevant) to 4 (Highly relevant), where ambiguity is the reverse criterion. Besides, the comment column for items was designed to collect experts’ opinions. To ensure ethical compliance, the validation form included detailed statements about data usage and privacy protection. All expert responses were handled with strict confidentiality, with individual feedback being anonymized and aggregated during analysis. Experts’ participation remained voluntary throughout the process with the option to withdraw at any stage without consequences. After three rounds of discussion and evaluation, the first round eliminated three items that were duplicates and did not quite fit the respective dimensions (AT5, AT6, and SE5) as well as modified some items’ wording and logic. In the second round, three reverse items were modified (AT3, AT4, and CA6). Two items on which no consensus was reached (EMF 2 and SE2) were discussed in the third round. The expressions of these two items were modified in the third round to avoid overlapping content between them. Finally, 26 items were retained for the pilot study.

Following the collection and analysis of experts’ feedback, the Content Validity Index (CVI) was computed to assess the content validity of the 26-item pool. CVI, a quantitative measure of content validity, evaluates the extent to which instrument items effectively represent the intended construct (Polit and Beck, 2006). Two types of CVI are commonly calculated: the Item-Level Content Validity Index (I-CVI) and the Scale-Level Content Validity Index (S-CVI) (Lynn, 1986; Polit et al., 2007). I-CVI is computed as the number of experts giving a rating of either 3 or 4 (on a 4-point relevance scale) divided by the total number of experts, with an I-CVI of 0.78 or higher being considered acceptable when there are 6 or more experts (Lynn, 1986). S-CVI can be calculated either as the Universal Agreement among experts (S-CVI/UA) or as the Average of the I-CVIs (S-CVI/Ave). With 6–10 experts: I-CVI ≥ 0.78 is acceptable and S-CVI/UA ≥ 0.80 is acceptable (Polit et al., 2007). The final calculations yielded an I-CVI of 0.95 and an S-CVI/UA of 0.81. The results indicate that the scale has excellent content validity.

### 3.3 Scale pilot study

To ensure respondents’ clear understanding of scale items, face validity, which assesses whether a scale appears to measure what it intends to measure from respondents’ perspective (Hardesty and Bearden, 2004), was established through an online focus group session involving 10 target users, including undergraduate (7) and graduate students (3) who regularly use AI tools for English language learning. During the 1-h session, participants engaged in structured discussions about each item’s clarity, comprehensibility, phrasing, and logical organization to their AI-enhanced English language learning experiences. The interactive discussion allowed participants to collaboratively examine the appropriateness of terminology and authenticity of items. Participants also discussed potential ambiguities and suggested refinements based on their shared experiences with AI-assisted language learning. The interactive nature of the focus group yielded valuable collective insights that informed subsequent item modifications to enhance clarity and practical applicability.

### 3.4 Participants

Ethical considerations were addressed through an informed consent form presented at the beginning of the online questionnaire. The consent form detailed the study’s purpose, voluntary nature of participation, confidentiality measures, data usage protocols, and participants’ right to withdraw. Participants could proceed with the survey only after indicating their consent by selecting “yes.” A large sample size (*N* > 1,000) was targeted based on recommendations for structural equation modeling and factor analysis, where larger samples enhance statistical power and provide more stable parameter estimates (Wolf et al., 2013).

The data were collected from four universities of mainland China. The four universities were selected based on a random sampling method. This approach was primarily driven by pragmatic considerations, specifically research feasibility and accessibility (Porter, 1999). The research team had established cooperative relationships or direct access to participant pools within these institutions, which was crucial for the efficient administration of the questionnaire and ensuring a satisfactory response rate.

While the selected universities are located in four distinct geographical regions of China (Guangdong, Hunan, Lanzhou, and Taiyuan), providing valuable regional diversity, it is important to note that they do not constitute a representative sample of all Chinese universities. Therefore, any generalization of the findings beyond institutions with similar characteristics should be made with caution.

The use of random sampling for site selection is considered appropriate for the initial stage of scale development and validation as the primary objective is to gather data for exploratory analysis rather than to make broad population inferences.

Data collection is conducted through an online questionnaire platform,^1^ with the survey link distributed via various educational institutions’ online networks and social media platforms frequently used by English language learners. The data collection period spanned from mid-March to mid-April. Prior to data collection, an official permission letter for data collection was obtained from the target organization. A total of 1,430 data samples were collected initially. After rigorous screening, verification, and removal of data with missing values, 1,093 valid datasets were ultimately retained for analysis.

The study participants comprised a diverse sample of 1,093 individuals with a relatively balanced gender distribution (52.71% male, 47.29% female). The majority were undergraduate students (56.84%), followed by graduate students (20.02%) and working professionals (19.56%), while doctoral students represented the smallest group (3.58%). Regarding AI tool usage for English learning, nearly all participants reported regular engagement, with 44.9% using AI daily and 49.31% using it 3–5 times per week. Only a minimal proportion (5.14 and 0.64%, respectively) reported infrequent usage (see Table 2). This demographic profile suggests a tech-savvy cohort predominantly consisting of students and young professionals, highlighting the pervasive role of AI in English learning practices.

**TABLE 2:** Demographic characteristics of the data.

Variables	Category	Number	Percentage
Gender	Male	574	52.71%
	Female	515	47.29%
Stage of education	Doctoral students	39	3.58%
	Graduate students	218	20.02%
	Undergraduate students	619	56.84%
	Working professional student	213	19.56%
Frequency of using AI tools to assist English learning	Daily	489	44.9%
	3–5 times per week	537	49.31%
	1–2 times per week	56	5.14%
	Rarely or never	7	0.64%

### 3.5 Data analysis

The study engaged a total of 1,093 valid respondents, which was a one-time collection. The total sample was randomly split into two independent subsamples using a computerized randomization procedure. The total sample size was randomly divided into two groups in SPSS, one group numbering 479 for exploratory factor analysis (EFA) and the other totaling 614 for confirmatory factor analysis (CFA) (Worthington and Whittaker, 2006). According to Comrey and Lee (2013), the results were considered very good when the sample size used for EFA reached 500. The subsample of 479, close to 500, was utilized for EFA to determine the initial factor structure and evaluate the construct validity of CFS-AISEL.

The data analysis primarily consists of three stages. First, prior to conducting EFA, skewness and kurtosis values were assessed to examine whether the data met the normality assumption. All variables’ skewness and kurtosis values fell within the ± 2 range, indicating that the distributions were approximately normal (Brown, 2007). Next, EFA was conducted using SPSS Statistics 27. Kaiser-Mayer-Olkin (KMO) coefficient was obtained to test whether the sample size was adequate for factor analysis. The Bartlett Sphericity test result showed the suitability of data for factor analysis. The principal component analysis and the varimax rotation technique were utilized in the EFA. Then, Mplus 8.3 software was employed to conduct CFA. The cross-validation subsample of 614 was reserved for CFA using structural equation modeling (SEM) to validate the factor structure emerged from the EFA and evaluate model fit indices, ensuring the robustness and replicability of the scale’s psychometric properties (Worthington and Whittaker, 2006).

### 3.6 Exploratory factor analysis of the CFS-AISEL

Prior to conducting EFA, the SPSS test results showed that the absolute value of skewness of all variables ranges between 0.623 and 1.190 while the absolute values of kurtosis range from 0.275 to 0.882. Hence, this indicates the data exhibit good normality, approximating a normal distribution. The KMO value of 0.926 and Bartlett’s test result (χ^2^: 6017.620, *P* = 0.000) as shown in Table 3 indicate that the sample size is adequate for the analysis. The Cronbach’s alpha reliability coefficient was 0.915, which indicated the CFS-AISEL scale has a high reliability.

**TABLE 3:** KMO and Bartlett’s test.

Test	Indicator	Value
KMO		0.926
Bartlett’s test of sphericity	Approx. chi-square	6017.620
	df	325
	Sig.	.000

As evidenced by Table 4, the five-factor structure comprising 26 items accounts for 64.344% of the total variance, indicating that these five identified factors collectively contribute nearly two-thirds of the total variance observed in the data. While scholarly perspectives on the threshold criteria for total variance explained remain diverse in the methodological literature, Field (2017) asserts that within exploratory research paradigms, a 60% explained variance is generally considered to be acceptable. This suggests that the current factor structure exceeds the commonly accepted methodological threshold for explanatory adequacy.

**TABLE 4:** Total variance explained.

Initial eigenvalues				Extraction sums of squared loadings			Rotation sums of squared loadings		
Component	Eigenvalues	Variance %	Total variance %	Eigenvalues	Variance %	Total variance %	Variance %	Total variance %	Eigenvalues
1	8.363	32.164	32.164	8.363	32.164	32.164	4.066	15.638	15.638
2	2.989	11.494	43.659	2.989	11.494	43.659	3.662	14.084	29.722
3	2.023	7.783	51.441	2.023	7.783	51.441	3.361	12.927	42.648
4	1.9	7.31	58.751	1.9	7.31	58.751	2.988	11.494	54.142
5	1.454	5.593	64.344	1.454	5.593	64.344	2.652	10.201	64.344

Source: Author’s own work.

**FIGURE 1:**
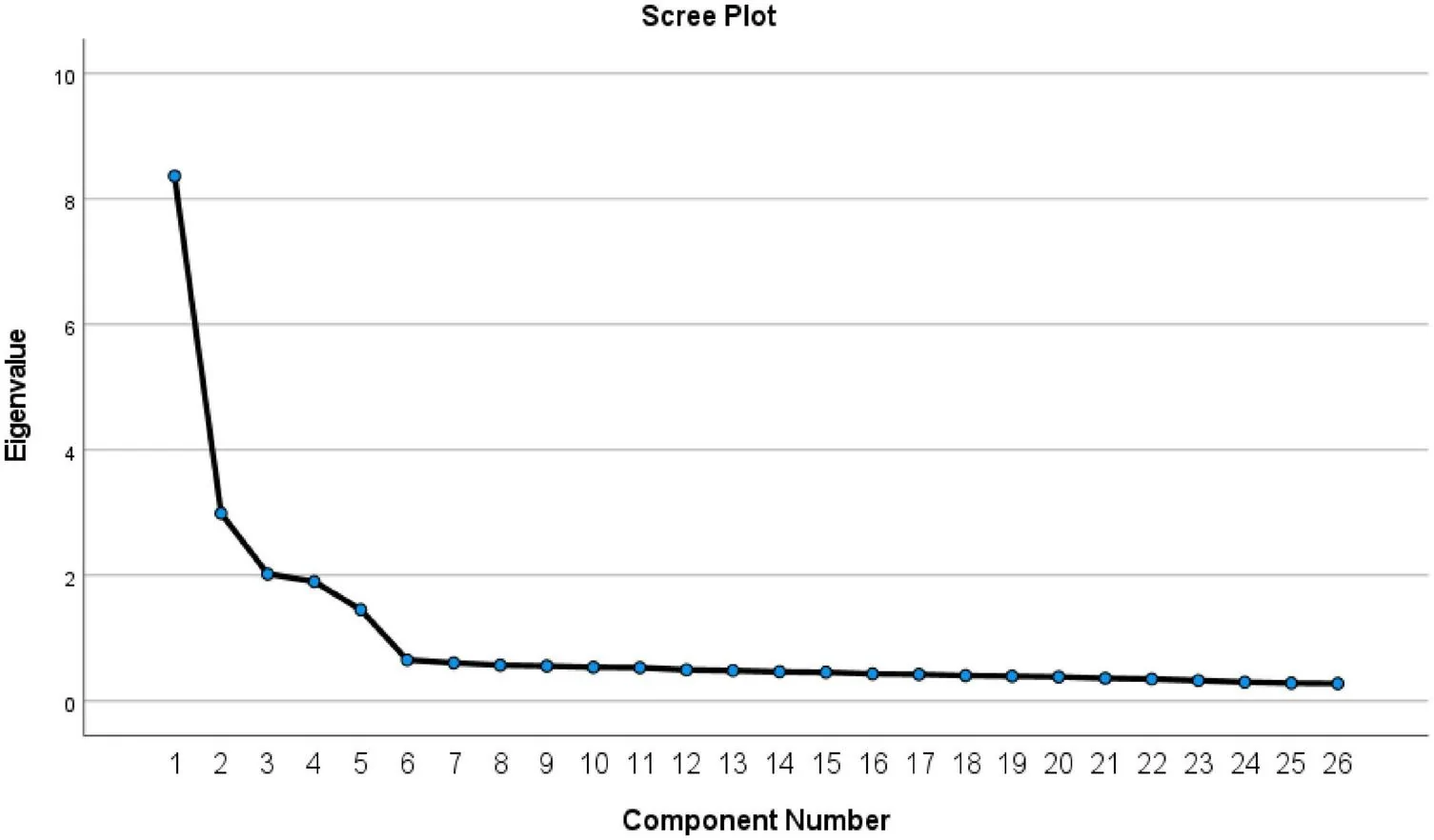
Scree-plot graph. Source: Author’s own work.

After rotation, the first factor contributes 32.164%, the second 11.494%, the third 7.783%, the fourth 7.31%, and the fifth 5.593% to the total variance. When the scree plot is analyzed, the line slope became horizontal after the sixth point (see Figure 1). Following established methodological protocols for dimensional extraction, an EFA was conducted. Based on the results of the scree plot and eigenvalues, a five-factor solution was retained, which is consistent with established psychometric criteria (e.g., Cokluk, 2026). This five-factor structure thus confirms the hypothesized dimensional composition of the scale.

The factor loadings of the scale items are presented in Table 5. The scale items’ factor loadings were distributed across 5 sub-dimensions. In the first dimension, factor loading values exhibited a range of 0.757–0.78 while the second dimension demonstrated values between 0.723 and 0.759. The third dimension displayed factor loading values from 0.752 to 0.798, with the fourth dimension showing values between 0.717 and 0.761, and the fifth dimension presenting values ranging from 0.718 to 0.784. Hair et al. (2010) asserts that factor loading values exceeding 0.7 indicates strong explanatory power of variables with respect to factors. Upon analyzing the items’ contributions to common variance, it was determined that all items demonstrated explanation rates meeting the required threshold. Thus, all items exhibited loadings greater than 0.7 on their respective factors with no significant cross-loadings. This indicates a robust correspondence between individual items and their extracted factors, eliminating the need for item removal. The corrected item-total correlation coefficients of the items ranged between 0.432 and 0.607, demonstrating that all items exhibit strong consistency with the overall scale.

**TABLE 5:** Factor loadings and corrected item-total correlations.

Items	Component					Corrected item-total correlation
	Factor 1	Factor 2	Factor 3	Factor 4	Factor 5	
CA1		0.751				0.468
CA2		0.723				0.443
CA3		0.759				0.481
CA4		0.757				0.432
CA5		0.755				0.545
CA6		0.757				0.446
AT1					0.784	0.507
AT2					0.778	0.489
AT3					0.718	0.53
AT4					0.781	0.524
SE1			0.791			0.549
SE2			0.798			0.55
SE3			0.762			0.551
SE4			0.752			0.583
SE5			0.769			0.547
EMF1	0.761					0.595
EMF2	0.766					0.593
EMF3	0.772					0.598
EMF4	0.78					0.607
EMF5	0.757					0.589
EMF6	0.776					0.604
EF1				0.761		0.477
EF2				0.759		0.457
EF3				0.748		0.492
EF4				0.717		0.432
EF5				0.748		0.461

CA, Cognitive adaptability; AT, Ambiguity tolerance; SE, Self-efficacy in AI interactions (SE); EMF, Emotional flexibility; EF, Ethical flexibility. Source: Author’s own work.

### 3.7 Confirmatory factor analysis of the CFS-AISEL

CFA was conducted on cognitive flexibility scale items to confirm the five-factor structure obtained from EFA with first-order and second-order multifactor models. CFA was performed by Mplus 8.3 and a subsample of 614 was used for cross-validation. Table 5 shows that the analysis focuses on examining the first-order multifactor model. The fit of the indices embraces several indicators: χ^2^, df, χ^2^/df, comparative fit index (CFI), Tucker-Lewis index (TLI), root mean square error of approximation (RMSEA), and standardized root mean square residuals (SRMR). Kline (2023) stated the aforementioned indicators provide adequate statistical reporting for research purposes. Moreover, according to Brown (2006) and Hair et al. (2010), model fit can be considered satisfactory when CFI and TLI values are greater than or equal to 0.90, while SRMR and RMSEA values are less than or equal to 0.05. In terms of the χ^2^/df index criterion, the analysis revealed an excellent model fit of the first-order model in Table 6. Other values also demonstrate excellent fit indices with χ^2^ = 391.772, df = 289, CFI = 0.987, TLI = 0.985, RMSEA = 0.024, and SRMR = 0.028.

**TABLE 6:** First-order CFA of the CFS-AISEL scale (with fit indices).

Fit index	Values	Evaluation
χ^2^	430.174	
df	294	
χ^2^/df	1.463	Excellent
CFI	0.983	Excellent
TLI	0.981	Excellent
RMSEA	0.027	Excellent
SRMR	0.041	Excellent

Source: Author’s own work.

Figure 1 presents the analysis diagram, illustrating both the item coefficients and inter-factor relationships. The obtained values demonstrate data-model fit, confirming that the structure identified through EFA for the CFS-AISEL is validated by the first-order multifactor model.

The values for the second-order level CFA results are presented in Table 7. These values also demonstrate excellent fit indices with χ^2^ = 430.174, df = 294, CFI = 0.983, TLI = 0.981, RMSEA = 0.027, and SRMR = 0.041.

**TABLE 7:** Second-order CFA of the CFS-AISEL scale (with fit indices).

Fit index	Values	Evaluation
χ^2^	391.772	
df	289	
χ^2^/df	1.356	Excellent
CFI	0.987	Excellent
TLI	0.985	Excellent
RMSEA	0.024	Excellent
SRMR	0.028	Excellent

Source: Author’s own work.

**FIGURE 2:**
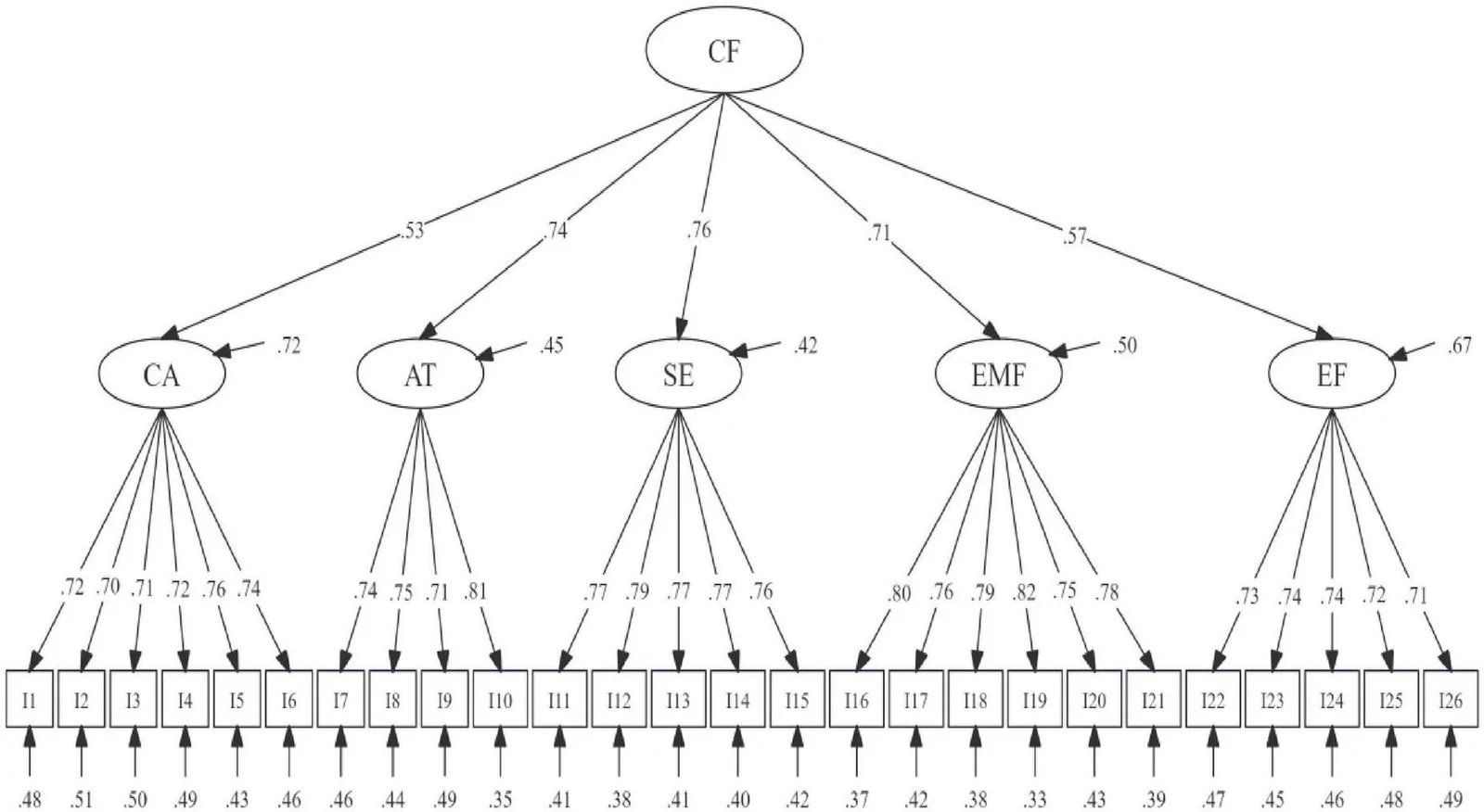
Second-order CFA of the CF. CA, Cognitive adaptability; AT, Ambiguity tolerance; SE, Self-efficacy in AI interactions (SE); EMF, Emotional flexibility; EF, Ethical flexibility. Source: Author’s own work.

**FIGURE 3:**
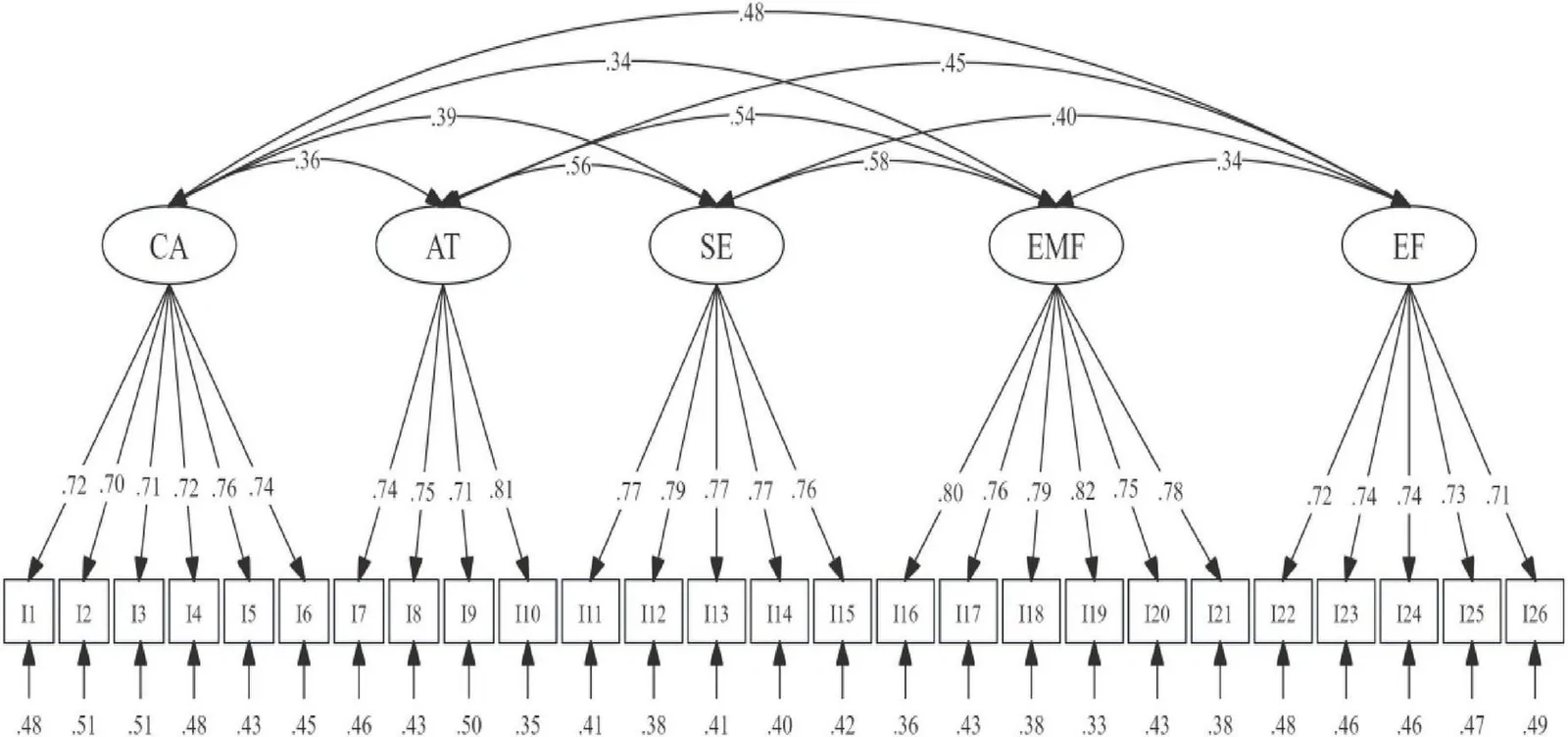
First-order CFA of the CF. CA, Cognitive adaptability; AT, Ambiguity tolerance; SE, Self-efficacy in AI interactions (SE); EMF, Emotional flexibility; EF, Ethical flexibility. Source: Author’s own work.

Cognitive flexibility demonstrates positive associations with its underlying sub-dimensions in Figures 2,3. Brown et al. (2015) suggests that loadings above 0.50 are considered meaningful in CFA models. The path coefficient between cognitive flexibility and cognitive adaptability is 0.53, while cognitive flexibility and ethical flexibility is 0.57. Besides, Kline (2023) notes that path coefficients above 0.70 represent strong and substantively meaningful relationships in hierarchical factor models. As shown in Figure 2, the path coefficient between cognitive flexibility and ambiguity tolerance is 0.74, between cognitive flexibility and self-efficacy in AI interactions is 0.76, and between cognitive flexibility and emotional flexibility is 0.71. Thus, the second-order multifactor model validated the structural framework previously identified through EFA for CFS-AISEL scale.

## 4 Discussion

This study aimed to develop the Cognitive Flexibility Scale for AI-Supported English Language Learning (CFS-AISEL). The scale was developed through a literature synthesis to identify initial dimensions and items, followed by an expert focus group to refine and finalize the item pool. A total of 1,093 samples were randomly divided into two subsamples: 479 cases were subjected to EFA using SPSS while 614 cases underwent CFA via Mplus. The EFA results revealed that the CFS-AISEL scale comprises five dimensions and 26 items, demonstrating structural coherence and theoretical alignment with the cognitive flexibility construct in AI-enhanced language learning contexts. The eigenvalues of the factors as a result of EFA were found to be 32.164% for the first factor, 11.494% for the second factor, 7.783% for the third factor, 7.31% for the fourth factor, and 5.593% for the fifth factor with the fifth-factor structure explaining 64.344% of the total variance. The results of the first-order and second-order CFA analysis revealed excellent fit models with χ^2^ = 391.772, df = 289, CFI = 0.987, TLI = 0.985, RMSEA = 0.024, and SRMR = 0.028 (first-order multifactor model) and with χ^2^ = 430.174, df = 294, CFI = 0.983, TLI = 0.981, RMSEA = 0.027, and SRMR = 0.041 (second-order multifactor model).

The dimensions of cognitive flexibility identified in this study align robustly with theoretical foundations established in prior literature (Deliu and Moldovan, 2026; Ionescu, 2017; Zuboff, 2019). Notably, the centrality of adaptability as a core component resonates with seminal work by Martin and Rubin (1995), who positioned adaptability as an essential pillar of cognitive flexibility, and Ionescu (2012), who further conceptualized strategic adaptation as a critical mechanism for navigating dynamic problem-solving contexts. Moreover, the inclusion of self-efficacy as a key dimension is empirically grounded in its well-documented role in bridging cognitive flexibility to behavioral outcomes (Lent, 2016; Yildiz Durak, 2023).

According to Bandura (1997), self-efficacy provides the motivational boost and confidence needed to move from flexible cognition to effective implementation (Andreassen et al., 2025; Martin and Rubin, 1995; Stajkovic and Luthans, 1998). Established role of self-efficacy in linking cognitive flexibility to behavioral enactment supports the scale’s construct validity, ensuring that it is consistent with established systems and promotes the complete operationalization of cognitive flexibility in psychology and education sectors.

This study deviates from the current scales (Martin and Rubin, 1995; Dennis and Vander Wal, 2010; Johnco et al., 2014; Wang and Jou, 2023) by including tolerance for ambiguity, which was theoretically consistent with the need to adapt to uncertain or novel demands (Ionescu, 2012). The most distinct ideas, however, lie in the addition of emotional flexibility and ethical flexibility. These dimensions emerged empirically to address context-specific demands of AI-supported language learning environments. As highlighted by structures on adaptive ethics in technology-integrated learning (Ionescu, 2012; Zuboff, 2019), ethical flexibility reflects the ability to navigate social dilemmas natural to AI-supported interactions. Emotional flexibility, likewise, captures the dynamic regulation of affective states required to sustain connection with AI systems’ unpredictability, resonating with an evolving conversation on human-AI emotional co-regulations (Namaziandost and Rezai, 2024; Shin, 2024). Ethical flexibility, a vital component of cognitive flexibility in AI-supported learning environments, enables learners to navigate ethical dilemmas by contextualizing judgments, balancing competing values, and dynamically adapting principles—key to managing academic integrity, developing AI ethics literacy, and resolving cross-cultural tensions in resource use, while developing awareness to algorithmic biases and data privacy (De Angelis et al., 2023; Iskender, 2023; Mbalaka, 2023; Nnorom, 2025; Pokkakillath and Suleri, 2023).

## 5 Limitations

There are several limitations in this study. First, the regional scope, while encompassing four representative provinces in each region, notable variations in EFL education quality, resource distribution, and sociocultural contexts across these regions could restrict the broader applicability of the findings to China’s heterogeneous student population, which may not fully capture the nationwide diversity of EFL learners. Second, urban-rural disparities in educational settings may not have been fully accounted for. A sample predominantly drawn from urban schools risks underrepresenting the unique experiences of rural EFL learners, particularly their limited access to AI-integrated learning tools. Urban students’ greater familiarity with advanced educational technologies, for instance, might introduce response biases when compared to their rural counterparts, potentially influencing scale interpretations.

To address these considerations, future research could further validate the ChatGPT literacy scales proposed in this study across varied sociocultural and educational contexts. As AI technologies continue to advance and their role in language education expands, subsequent iterations of the scale would likely benefit from incorporating additional dimensions and refined items to align with evolving pedagogical practices and technological innovations. Furthermore, this study is expected to become a study that establishes a basis for future studies exploring the role of cognitive flexibility. Cognitive flexibility, as a multidimensional construct, can assume distinct roles in theoretical and empirical frameworks depending on research objectives. It may act as a mediator to explain mechanisms (e.g., linking multilingual exposure to enhanced creativity via improved adaptive thinking), serve as a moderator to conditional relationships (e.g., amplifying the benefits of AI tools for learners with higher flexibility), or function as an outcome variable influenced by interventions such as cognitive-behavioral therapy or cross-cultural experiences. In complex models, it might simultaneously mediate and moderate pathways (e.g., mediating team diversity’s impact on innovation while being moderated by leadership styles). These versatile roles underscore its centrality in domains ranging from education and mental health to organizational behavior, offering a dynamic lens to examine how individuals adapt to cognitive, technological, and sociocultural challenges.

## 6 Conclusion

In conclusion, the Cognitive Flexibility Scale (CFS), comprises five distinct components: cognitive adaptability, tolerance for ambiguity, self-efficacy in AI interactions, emotional flexibility, and ethical flexibility. As a five-point Likert assessment instrument, the scale consists of 26 items that demonstrate solid psychometric properties. Importantly, CFA revealed that things constantly mapped to their theoretical realms while demanding construct validity testing demonstrated that the scale’s reliability coefficients significantly exceeded established levels. The convergent and discriminant validity provided more evidence for the scale’s ability to distinguish between cognitive flexibility and related constructs. These findings collectively position the CFS as a reliable and valid instrument for assessing cognitive flexibility in the psychology and educational sector, offering researchers and practitioners a multidimensional tool to evaluate this critical adaptive capacity. Future studies should explore cross-cultural validation and predictive validity in real-world decision-making scenarios.

## Data Availability

The raw data supporting the conclusions of this article will be made available by the authors, without undue reservation.

## Footnotes

1
www.wjx.cn
